# Did Orpheus have stage fright? To oneself, to the other, to the transcendent: steps for coping with music performance anxiety

**DOI:** 10.3389/fpsyg.2025.1706198

**Published:** 2025-12-17

**Authors:** Natalija Šimunovič

**Affiliations:** Academy of Music, University of Ljubljana, Ljubljana, Slovenia

**Keywords:** music performance anxiety (MPA), musical self-concept, musical manifestation, transcendence, Orpheus

## Introduction

Orpheus, as an archetypal model of musical performance, enters our cultural consciousness not only as an exemplary performer but also as a figure whose fleeting glance into the visible shattered the magic of creation. Through this metaphor, for three millennia we have (re)interpreted the deeper projection of the conflict between the artistic idea and the performer's inner tension (Partridge, 2014; Blanchot, 1981). The performer's inner tension is partly framed by the constructs of stage fright (Steptoe, 1989) and music performance anxiety (MPA; Kenny, 2011), which empirical research often links to impaired stage performance (Chan, 2011). MPA is consistently associated with lower self-efficacy (Bandura, 1997; Zarza-Alzugaray et al., 2020) and reduced self-esteem (Rosenberg, 2015/2015, Jiang and Tong, 2024), as well as heightened self-monitoring and threat appraisal (Kenny, 2011; Brooks, 2014), perfectionistic concern (Spahn, 2015), and attentional narrowing with a consequent loss of the “big picture” of performance (Clarke et al., 2020). Such processes hinder the optimal flow of performance, understood as a balance between challenge and skill (Csikszentmihalyi, 1990).

Because the myth portrays Orphic performance as technically flawless, the motif of lapse directs us to a dimension that anchors peak performance beyond mere technical execution (Privette, 1983). Privette supplements the apex of creative activity with the notion of peak experience, which brings self-transcendent qualities into performance. Precisely the moment when Orpheus “loses the transcendent thread” entails a breakdown of the performance and its meaning—a motif we subsequently use as a philosophical key for a cautious, empirical dialogue with the analogy of the myth. We ground this dialogue in findings indicating that mindfulness and related contemplative practices may facilitate a shift from an anxious mode to a more optimal performance state (Czajkowski et al., 2020; Paese and Egermann, 2024; Paese and Schiavio, 2025), while understanding the transcendent dimension as both performing at the height of one's abilities and experiencing a sense of belonging to something beyond the individual (Bernard, 2009; Jääskeläinen, 2022).

In this article, we argue that, given the overarching motive of musical creation, approaches to MPA should be extended beyond the *I/self* and *Me/others* dimensions to the transcendent dimension, focusing on contexts such as rituality, community, valuation, mindfulness, and the search for higher meaning in music performance. In education, this means intentionally cultivating practices in which the performer, alongside balance with self and audience, also seeks a universal meaning—the third vertex of our triangle—as a protective ring against MPA.

## Self-concept and musical manifestation

“… *not conceptual speech, but music rather, is the element through which we are best spoken to by mystical truth*.” (James, 1902/2004, p. 328)

In the above quotation, the philosopher and psychologist William James uses the word *mystical* in relation to music. In strictly empirical approaches—focused on quantitative testing and applicability—such terminology appears only rarely. Instead, it is more often invoked in philosophy and the psychology of religion, or replaced with more measurable concepts such as *transcendental experience, absorption, flow*, or *aesthetic chills*. Yet it was precisely William James (James, 1890) who laid the foundations for an empirical investigation of the unseen but essential source of human meaning—the soul, a concept already central to Plato's philosophy (Plato, 2004). In *The Principles of Psychology* (1890), James ascribed to this entity a stream of directed consciousness that one devotes to oneself. In this way, he established the concept of *self* , thereby relocating the domain of the soul, or the *thinking self* (Descartes, 1966), into the realm of cognitive interpretations of personality. This marked the beginning of a systematic psychological delineation of the individual, which (James 1890), (1902) framed through bodily, material, social, and spiritual dimensions of perception. Were he writing today, he would likely call out the persistent elusiveness that clings to the spiritual stratum within self-concept research (see Hattie, 2003).

Since the 1980s, the musical self-concept and, consequently, musical identity have attracted growing attention among music psychologists (Asmus, 1986; Frith, 1981; Hargreaves, 1986; Schmitt, 1979; Svengalis, 1978). The most comprehensive account was provided by Maria Spychiger, who explained it from a philosophical and psychological perspective (Spychiger, 2010; Spychiger and Hechler, 2014; Spychiger, 2017b) as well as from a music education perspective (Spychiger, 2017a, 2021), while also describing its empirical basis and measurement procedures (Spychiger et al., 2009; Spychiger, 2017b).

Based on the ideas, perceptions, and evaluations reflected in an individual's diverse understandings of their musical activities, the self-concept is divided into an **academic dimension** and a **non-academic dimension** (Shavelson et al., 1976; Spychiger et al., 2009). The academic dimension encompasses domains of knowledge and skills such as singing, playing an instrument, auditory abilities, and composition. The non-academic dimension comprises physical, emotional, social, cognitive, and spiritual aspects of the musical self-concept. According to Spychiger, the musical self-concept represents a non-hierarchical and multidimensional psychological construct of the individual, which, during adolescence—in both social and developmental terms—evolves into musical identity. This psychological construct connects personal perceptions with social roles and acts as a key regulator of musical development and experience (MacDonald et al., 2002).

The self-portrait of the musician, as captured by the MUSCI (Musical Self-Concept Inquiry) Scale (Fiedler and Spychiger, 2017), extends beyond the emotional, social, or ability-related aspects of previously established measures, as it enables an understanding of musical functioning through the spiritual and transcendent horizon of artistic experience. The scale includes methodological items such as: “*For me, making music is a special kind of prayer; I make music in order to feel the divine; I like to make music which promotes spiritual experience; With my music I can elicit change in people*.” (Spychiger, 2017b)

**Figure 1:**
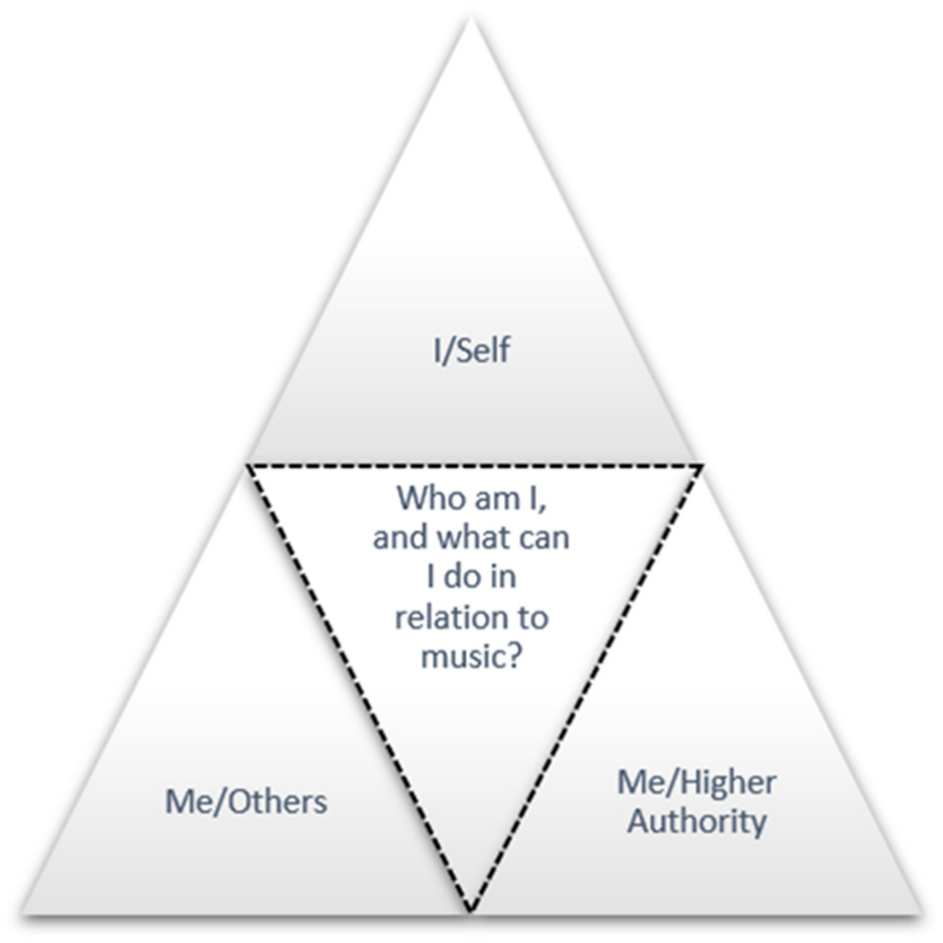
Vertices of the musical manifestation.

The MUSCI scale thus operationalizes an entity that is not the person him or herself, but rather the product of his or her relationship with the irrational (Spychiger, 2019). Such multifacetedness shapes musical manifestations that, in an individual's relationship (i), with him or herself (ii), with others, and (iii), with an entity that transcends or complements the individual and society, raise the fundamental question: Who am I, and what can I do in relation to music? (Spychiger, 2017b). Within this framework, internal reflection, interpersonal interaction, and a broader connection to the transcendent are formed, which—as shown in Figure 1—is illustrated by a triangular scheme of three vertices.

Let us imagine a child who, during instrument lessons, has learned to play a melody that he knows well—it is a folk song that his mother sang to him. When learning a melody on an instrument, he will face challenges related to his knowledge and skills, physical capacity, and emotional experience. This involves triggering cognitive processes that guide the understanding and processing of musical material, as well as social aspects that stem from interactions with the teacher, peers and audience. Learning and performing a simple song will thus provoke self-reflection, socially reciprocal dialogue, and the musical manifestation itself will also reflect a relationship to a higher entity—in this case, to national belonging, which transcends both the child and his perception of others. The melody, as a musical message, will take on the symbolic power of national belonging and music heritage, and the child will thereby assume the role of bearer of this important message. At the same time, the child will undergo a complex musical experience in which s/he will also articulate the spiritual purpose of creation.

In this triangular schema MPA is expected in areas where the burden exceeds the regulatory abilities of self-concept; empirical studies often perceive this as lower self-efficacy and/or lower self-esteem (Bandura, 1997; Chan, 2011; Dobos et al., 2019; Rosenberg, 2015/2015). When self-concept is stronger, it is easier to absorb stage pressure—higher self-esteem and especially musical self-efficacy are associated in research with lower MPA and better performance control (Castiglione et al., 2018; Jiang and Tong, 2024; Zarza-Alzugaray et al., 2020; Wang and Yang, 2024). This is also revealed by the symptoms that occur with MPA: emotions are exacerbated, the whole body reacts, cognitive resources are narrowed, and the social context intensifies judgment. However, the existing literature is dominated by a discourse in which therapy for the relief of MPA is framed in terms of *I/self* and *Me/others* relations, while the connection to the transcendent (e.g., Bernard, 2009; Jääskeläinen, 2022) has received comparatively less sustained attention.

## To the transcendent: Future steps for coping with music performance anxiety

In the *I/self* dimension of our triangular schema, MPA is managed with relaxation, gradual exposure, and cognitive behavioral therapy (CBT), and beta-blockers have been used as adjuncts to treat physical symptoms (Kenny, 2011; Kinney et al., 2025; Nicholl and Abbott, 2025; Spahn, 2015; Szeleszczuk and Fraczkowski, 2022). More modern CBT practices—especially Acceptance and Commitment Therapy (ACT)—do not just address symptoms, but also strengthen the relationship to one's own experiences and values. In a group or “coaching” setting (Clarke et al., 2020; Juncos and Markman, 2016) this brings us somewhat closer to the *Me/others* dimension. Psychodynamic relational approaches explicitly explore early relationships, transference, and the context of performance (Kenny et al., 2016), which directly affects the *Me/others* dimension. Guided mentoring and social support strengthen bonds with teachers, peers, and audiences and indirectly increase self-efficacy (Huawei and Jenatabadi, 2024), which is associated with lower MPA; brief reappraisal strategies—e.g., “rewrite anxiety into excitement”—are also helpful before and during a performance (Brooks, 2014; Jamieson et al., 2013; Zarza-Alzugaray et al., 2020; Uphill et al., 2012).

We enter the transcendent dimension of performance through the latent analogy of Orpheus and his creative identity—an archetype that opens the cracks of the sacred within the secular in music (Partridge, 2014). He represents the ideal of artistic interpretation, since his singing and playing of the lyre were so powerful “…*that even the wild animals were stilled, trees and stones moved to the rhythm of his music…*” (Apollodorus, 2008, 1.3.2; Ovid, 2004, 11.1–5). Yet Orpheus cannot fully own his artistic potential, which is woven into his musical identity as a singer, instrumentalist, composer, musical mentor, therapist, and liturgist. The mythical origin of Orpheus' excellence is attributed to his mother, the goddess Calliope, and the god Apollo, who gave him the lyre and taught him to play (Apollodorus, 2008, 1.3.2).

With this premise, how should we view contemporary society, which is far from unified in its worship of God or deities, and which can no longer be addressed en masse with either the *Matthew Passion* or gospel? We thus witness new forms of belief as believers—understood here as devotees ascribing sacred significance to emblematic figures—venerate so-called secular saints (Rojek, 2001). On the basis of a subjective and transcendent experience with, say, Whitney Houston, these devotees then undertake contemporary pilgrimages to digital shrines, the grave, and memorial sites—through which the singer's widely mediated vocal presence functions as a spiritual authority and an institution outside ecclesial settings, especially online (Chidester and Linenthal, 1995; McCutcheon et al., 2002). The contemporary spiritual landscape therefore offers, in addition to the sediments of traditional religions and the confusion and dilemmas related to them, a range of other powerful ideas, encrypted and fragmented within secular entities (Partridge, 2014). From this perspective, spirituality should not be sought solely within religious contexts and acts of worship, but also in secularized spiritualized entities that are not themselves human yet exert significant influence on humans (Spychiger, 2019).

Secularized sources of veneration may manifest as political ideas or movements, but they can equally be attributed to entities such as recording contracts, the position of concertmaster, or even a self-idealized status as a virtuoso, conceived as a network of spiritual purposes and goals. This amounts to privileging certain cognitive concepts of music manifestation over others. If we consider further the individual who idealizes personal virtuosity, career ambitions, or technical perfectionism, research shows that such factors are associated with greater vulnerability to MPA (Castiglione et al., 2018; Dobos et al., 2019; Kenny, 2011).

The analogy we deepen with Orpheus's lapse tells us the same: an inflated dimension of the self can lead to the disintegration of a holistic artistic identity. The excessive desire for earthly love—for Eurydice, or, in modern terms, the all-encompassing lust for success—conceals a creative trap. The ancient myth predicts the artist's disintegration, and the fall of his mission; our reality, along with certain modern myths, points to similar fates of great performers.

It may therefore be a task for future researchers to strengthen this premise and treat MPA as a symptom of a broader imbalance, as intimated by Leech-Wilkinson (2018), Skoogh and Frisk (2019), and Perdomo-Guevara (2014), rather than as a standalone disorder (see Fernholz et al., 2019; Sousa et al., 2016) that burdens us with a series of superficial, unmanageable ailments.

## Music performance anxiety as a symptom of asymmetry of the vertices of musical manifestation

In the field of musical identity—as a developmental and social consequence of the musical self-concept—since 2002 a range of musical practices has been reconsidered through the lens of personal and social coherence, reciprocity, and meaningfulness (MacDonald et al., 2002). We identify the latter as emotional wellbeing (Saarikallio, 2017), economic and personal musical synchronicity (Gaunt et al., 2021), optimal musical development (Taruffi, 2017; Evans and McPherson, 2015; Lamont, 2017; O'Neill, 2002), and activities in which a therapeutic (Bradt et al., 2013) or humanitarian and connecting meaning is increasingly indicated (Hess, 2019). Musical identity cannot therefore be the property of an individual, but is created by the common dynamics of coexistence in which the needs of society are outlined. Thus, in the field of education and performance practices, Lukowska (2024) emphasizes the triple mission of the musician as composer, performer, and teacher, and warns that focusing solely on virtuosity creates discrepancies in professional identity and pedagogy. These are reflected in the literature as phenomena of vulnerability and conflict in the identity of the musician (Pellegrino, 2009).

Hargreaves et al. (2003) also focuses on the individual's identity as a regulatory instrument in musical activity, which we must take into account in educational processes as a concept of socially meaningful development of the musician. Such global concepts may also offer a kind of “release from suffering” for those who, even during learning, free themselves from self-perceptive patterns that produce tension rather than wellbeing, and self-absorption rather than creative community. In this way, the “uncalled” will not be unduly burdened by excessive technical overemphasis, constrained bodily expression, or social categories that fail to address their musical self. However, a mere structural step toward oneself and others does not yet bring the full meaning of music performance. The “gods of the underworld” must be addressed with an elaborate system of values and an equivalent creative language, which is not necessarily Orpheus' artful melody but can be a simple folk song that elevates national consciousness. Encouragingly, studies attribute the transformation of anxiety into creative power to transcendent experiences. Alberici (2004), Bernard (2009), and (Jääskeläinen 2022) report that musicians experience such moments as healing and at times even “divine,” as they transcend not only performance stress but also the burdens of everyday life. One participant defined transcendent performance as “*the experience of rising above normal physical and mental fears and concerns to a peak experience that is remembered and sought after again and again*” (Alberici, 2004, p. 22).

## Discussion

With full respect for Orpheus as a possible historical figure (Wroe, 2011) or as an incarnated myth that has left the world priceless artifacts, his story should not be trivialized by pursuing a literal answer to our hypothetical title question. We can, however, understand the turning point—where the mythical figure failed to complete his performance—as an expression of the fragility of his self-concept (Blanchot, 1981), in which stage fright can also be understood as a vulnerability within the realm of narcissism (Gabbard, 1983; Nagel, 2018).

We have therefore examined the archetypal ideal of the musical performer as a prototype of performative (im)perfection that unravels due to identity incoherence (Blanksma, 2024). To that end, we have clarified the internal robustness of the performer's musical manifestation, in which Orpheus embodies, above all, the third vertex—the ritual, the religious, the transcendent (Spychiger, 2019). This dimension is by no means disappearing from our everyday lives. On the contrary, within secular conditions (Partridge, 2014), it grows more colorful, more intricate, and more contested in value. It also shapes musical performance, which, in future research, should approach the emergence of MPA not merely as an individual symptom but as a socially shaped phenomenon (Brooks, 2014; Herman and Clarke, 2023). We hold that its regulation is offered by the self-concept (Hargreaves et al., 2003) and by the formation of musical identity as a shared capital of the individual and society, connected with the higher meaning of musical manifestation.

We must enter that manifestation as beings who have been given a voice, a lyre, and Eurydice—knowing that nothing in it, except the song that, in that very moment, resounds across the living and the dead, is immortal, immutable, or tangible.
